# Economy a Duty

**Published:** 1878-08

**Authors:** 


THERE never has been a time in our nation's history, when the obligation on all classes has been so urgent to economize in every possible direction, in food, in dress, in rents and in every minor article of expenditure. The rich should practice economy for the sake of setting a good example to those in more moderate circumstances. The wives and daughters of half our citizens are demented; they no more know the value of money than an equal number of Egyptian mummies; this may seem at first sight a most extravagant statement; but it is short of the truth. A mummy knows nothing; a live woman of our time has arrived at a state of mind by false knowledge, which leads to more pernicious results, than if she knew nothing at all. A mischievous argument has been presented by some, that the rich should spend as much as possible, and thus, not only give employment to those who are willing to work, but by a lavish expenditure in the way of dress, make importations from abroad more necessary, and thus the income of the government will be increased through the Custom-House. But it is forgotten that gold must be sent to pay for these goods, and by this incessant drainage the amount of our coin becomes less and less, and the price of every article of consumption becomes greater and greater. But suppose there were no importations, foreign countries being obliged to send for millions of dollars' worth of wheat and corn and meal and flour, would have to send also, these millions in gold, making it more abundant every month, instead of its becoming more and more scarce. The more gold there is, the less every article of consumption costs; the less would be the need of hurry and exposure, and over-work of body and mind, which things kill multitudes prematurely every year; while more time would be afforded for rest, for rational enjoyment, and for that mental and spiritual and social cultivation which so add to human elevation and human happiness.

If the times become better present economies will injure no one; whereas, if they become worse, the people will be better prepared to meet them. The safe and experienced sailor anticipates the storms of the sea; and they are wisest who look forward to clouds and darkness and tempests in the business future. Economies should begin in dress and food and house and servants. Old garments should be patched

As to the item about servants, it is the greatest shame and disgrace of our people, especially in New-York. We know a family of five persons which keeps four servants. Another of three, keeps three servants; some families, strictly private, have seven, eight, or nine helps. If this over-supply of servants ended simply with the increased expenditure of the particular family, the evil would not be so great, in the few cases in which the hire and board of these retinues are not paid eventually by other and more honest and industrious people. But it is notorious, that generally, such persons fail and their creditors are the real sufferers. The really rich of New-York, those who have been wealthy for a generation or more, are the only persons, as a class, who do practice a wise economy. They do it as a pleasure, arising from an honorable conviction of the justice and right and prudence of their course, and for the assurance which it gives them of a continuance of a comfortable competence in the long years of the future.

But this extravagant supply of servants has a pernicious effect on the servants themselves; they become inevitably more and more idle, careless, inattentive, impertinent, and wasteful; and when these qualities have arrived at an unendurable pitch, they are sent away, and then they impose themselves on less aspiring families, to annoy them by their worthlessness; and in a few years they go down lower and lower in the scale of efficiency, are more and more unemployed, their scanty earnings become exhausted in the miserable hovels in which they board; miserable enough, as all ladies have learned who attempt to hunt them up in answer to advertisements in the papers. Some of the places where cooks and chambermaids board while they are getting places, are not fit for the habitation of horned cattle; a good farmer would not keep his horse or his cow in such rickety, unventilated, and blackened apartments, situated as they generally are, in the distant, filthiest, and most noisome streets and alleys in the whole metropolis. And yet, when these same persons are introduced into a respectable dwelling, they assume the airs of duchesses and queens. They can't use brown sugar in their coffee, because it gives them the headache. They won't touch any other bread than that which is cut fresh from the loaf at the time they are wanting it; while the slices left at the family table of to-day, if not thrown into the ash-barrel, or given to some begging cousin or acquaintance, are placed on the family table for the next meal. None but the costliest tea will "agree" with their delicate stomachs, and this is made so strong, that in order to be able to drink it, they saturate it with loaf-sugar. Unless they are closely watched on washing days, their own clothing first passes through the laundry; is first hung out to dry, and that too in the sunniest places in the yard; while in the starching process of skirts, etc., their own are made as stiff as pasteboard, and in every respect have the preference. Such impertinences as these, the less resolute of our wives have to endure, and in consequence, are kept in a state of irritation and fretfulness and anxiety which wastes the strength, ruffles the temper, sours the disposition, and makes housekeeping a penance instead of a happiness.

Economy in house-rent is becoming more and more a necessity, and it is greatly to be regretted that it is not more common for two families to live together and divide this expense and that of servant-hire. In many families there is really no use for an "up-stairs girl" or chambermaid, or waitress, if the "door-bell" had not to be attended to; and it is not much more trouble to cook for two families than for one, if they are well-ordered ones. In this way, better wages can be afforded and better servants can be had; thus not only will the board of two servants be saved, but the comfort of having faithful and competent ones, will be worth more than money itself.

It is often said and generally believed, that two families can not live together under the same roof. This is a great mistake; when two women can not live in the same house in comfort and peace and social enjoyment, it is because neither of them have any sense of a practical kind, and have very little religion or good principle of any sort; it is because they have no true religious principles; have been raised in vulgarity, or have had for mothers, persons who were unworthy of the sacred and blessed designation. Is a woman no better than a cat or a dog, that she can't dwell under the same roof with another in peace and harmony, with the common end of having a better table, better servants, a more lively household, at less expense and labor and anxiety and care, than if each family lived in a separate dwelling? We can not harbor such an opinion of our wives and daughters for a single moment. We have visited a house several times this last winter, one of the choicest and most truly elegant in this city, in which lived three distinct families, with two sets of children, from a year old to seven, of boys and girls; each family was abundantly able to keep their own establishment, each having houses of their own; the washing was put out; one housemaid and one cook, both good, did all the work for these three families throughout the whole winter; there was not the slightest jar or unpleasantness, even among the servants; the children, we were told, were like brothers and sisters of the same family; and not a single unkind word was ever known to have passed between them; while a more abundant table, better prepared, and a neater and better kept house is seldom seen in this great metropolis. We personally know two families, both rich, who have lived in the same house for three years, and are as fast friends to-day as when first they cast their fortunes together; not so much for saving as for social enjoyment. We know of another household, made up of three or four different families, all independent; and go there any day, live there any week, and you would not know but they were all of a common stock. Let any considerable number of families have the moral firmness, the high Christian principle, the wisdom and the patriotism, to unite and make combinations of this sort popular and honorable, and in three months there would be the inevitable result of fifty per cent diminution in house-rents; servant-hire would be largely less; first-class servants would be in greater demand; while the trifling and dishonest and unprincipled would be glad to get work for little more than their victuals and clothes, simply because half the number of helps could be dispensed with, and the highest wages could be afforded to those who were really competent, and were willing to give their whole time to their employers. If two families combine and give a really competent cook twelve dollars a month, the saving of food and comfort and health secured by that arrangement, as compared with two separate households, with six-dollar cooks, could not be easily estimated. What is the great hindrance of a social reform like this; one which would not only save millions of dollars annually to the hard-working men who have to earn these dollars, but would be a means of greatly adding to social enjoyment and domestic comfort and general elevation and enlargement of the spheres of thought? The only obstacle is the pride and ignorance and selfishness of the wives of the country. Not one husband or one brother in ten among the classes most needing it, would object to such an arrangement; the opposition would be almost wholly from mothers and marriageable daughters. A society organized with a view to bringing about a custom of this sort, would, in proper hands, do more good socially, religiously, and in a patriotic point of view, than nine out of ten of those of the later times.

Another plan, in some respects better, in others not so good, is that of having houses built, as in the older countries of Europe, having each story constructed so as to afford full accommodations for a whole family; these are called flats or floors; the highest are the healthiest and cheapest. But it would take years to make arrangements for this manner of living, as houses would have to be built especially for that purpose; the other plan could be carried into effect at once.

Another item of unnecessary extravagance of the times, is the imagined necessity of doing business in one part of the city and living in another. A clerk with six hundred a year must live up-town. A man, whose business is worth two thousand dollars per annum, must have his "store" down-town, and rent a house two or three miles away. Many of these ought to do business on the first floor and live up-stairs; the oldest and most honored merchants in our memories "did business" in this way, and have left names and fortunes behind them, which their children and grandchildren are still living to be proud of and to enjoy. The Paris Rothschild is said to live in the rear of the building in which he does his business.

Let our readers be assured that the purest and truest and highest patriotism of our times, is not the blatant cry of Unionism, liberty to all, free soil, and all that, but it is individual integrity and personal economy in their highest and strictest forms, carried out in every minutia of domestic expenditure.

There is another method of exhibiting a high patriotism, as a means of saving the national credit, and preventing a national and individual financial collapse; it is easily stated in a few words, to swear or affirm, in plain monosyllables: "From this good hour, I will not eat or drink or wear what does not grow in the land of my birth, the land I most love."

But we have too much apparent prosperity; fashion and folly and mad extravagance have too great a control over our people, to allow even the glimmer of a hope that such virtue can exist. Who is the maid or matron in New-York who will have the heroism of a Joan of Arc, and will step out of the ranks as the leader, in a cause so grand and glorious and good?

